# Dynamic changes in the gut microbiota of SPF Bama piglets during breast and formula feeding

**DOI:** 10.3389/fmicb.2025.1537286

**Published:** 2025-02-26

**Authors:** Chengcheng Zhang, Zhengjiang Liu, Huan Yu, Yuanyuan Shen, Lu Lu, Fanli Kong, Wei Sun, Xiaoyuan Wei, Long Jin, Liangpeng Ge, Bo Zeng

**Affiliations:** ^1^Key Laboratory of Livestock and Poultry Multi-omics, Ministry of Agriculture and Rural Affairs, Farm Animal Genetic Resources Exploration and Innovation Key Laboratory of Sichuan Province, College of Animal Science and Technology, Sichuan Agricultural University, Chengdu, China; ^2^State Key Laboratory of Swine and Poultry Breeding Industry, College of Life Science, Sichuan Agricultural University, Ya’an, China; ^3^Joint International Research Laboratory of Agriculture and Agri-Product Safety of Ministry of Education of China, International Joint Research Laboratory in Universities of Jiangsu Province of China for Domestic Animal Germplasm Resources and Genetic Improvement, Yangzhou University, Yangzhou, China; ^4^Department of Internal Medicine, Morsani College of Medicine, University of South Florida, Tampa, FL, United States; ^5^Key Laboratory of Agricultural Bioinformatics, Ministry of Education, Chengdu, China; ^6^Key Laboratory of Pig Industry Sciences, Ministry of Agriculture and Rural Affairs, Chongqing Key Laboratory of Pig Industry Sciences, Chongqing, China

**Keywords:** gut microbiota, pig breeding, breastfeeding, formula feeding, metagenomics, SPF Bama pig

## Abstract

The gut microbiota plays a crucial role in the growth performance, health status, and welfare of pigs. Breast milk is a key factor in the colonization of gut microbiota and the overall health of newborn piglets. With advancements in breeding technology, formula milk has been widely adopted as a substitute for breast milk. This study aims to investigate the effects of sow feeding (natural breastfeeding) and formula milk feeding on the gut microbiota of specific pathogen-free (SPF) Bama pigs. Using metagenomic sequencing technology, we analyzed 114 fecal samples to uncover the impacts of different feeding methods on gut microbial diversity, dominant microbial populations, metabolic functions, carbohydrate-active enzymes (CAZymes), and antibiotic resistance genes (ARGs). The results revealed significant differences in the structure and function of gut microbiota between the breast milk (BM) group and the formula milk (FM) group at day 21. The BM group exhibited higher gut microbial diversity compared to the FM group, along with more extensive metabolic functions at both the gene and species levels. Notably, the FM group demonstrated higher activity in galactose metabolism and glycan metabolism, particularly at day 21. Additionally, the FM group showed significantly higher levels of ARGs against glycopeptide antibiotics at days 21 and 28 compared to the BM group. This study also found that breastfeeding and formula feeding differentially regulate the metabolic activity of gut microbiota and the expression of related enzymes, which may have long-term effects on nutrient absorption and disease resistance in pigs. These findings provide new insights into how different feeding methods shape the gut microbiota of pigs and offer a scientific basis for optimizing feeding strategies and improving breeding efficiency.

## 1 Introduction

The gut microbiota plays a pivotal role in influencing the growth performance, health status, and overall welfare of pigs. It contributes to essential physiological processes, including nutrient digestion and absorption, immune regulation, and pathogen suppression (Patil et al., 2020; Mu et al., 2019). In pig husbandry, the early colonization of microbial communities within the gastrointestinal tract significantly impacts organ development and immune maturation in piglets. Newborn piglets initially possess an immature intestinal tract, which struggles to accommodate a high abundance of microbial colonization. However, as intestinal development progresses and functional improvements occur, the gut microbiota gradually matures and stabilizes (Wang et al., 2019; Mach et al., 2015). In practical breeding scenarios, sow’s milk serves as the primary source of nutrition and protective substances for neonatal piglets. As the exclusive dietary source during this early stage, it plays a crucial role in establishing the gut microbiota. The microbiota, along with various components found in breast milk, may modulate the infant’s immune system by promoting the growth of beneficial bacteria while inhibiting harmful pathogens, thereby influencing immune cell development and function (Jang and Kim, 2022; LeMaster et al., 2023). For instance, oligosaccharides, a key component of breast milk, stimulate the growth of beneficial bacteria such as bifidobacteria while preventing pathogenic bacterial adhesion, thereby positively influencing gut microbiota establishment and immune functionality (Le Doare et al., 2018; Liu et al., 2019). However, advancements in breeding technology have led to increased litter sizes and maternal health issues, which may prevent newborns from accessing adequate amounts of breast milk. Consequently, alternatives such as formula milk, designed to mimic breast milk composition, have been developed to meet the nutritional needs of piglets (Chong et al., 2022). Formula feeds aim to replicate the nutritional profile of breast milk while incorporating probiotics, prebiotics, and other functional ingredients intended to regulate gut microbiota dynamics. Despite these efforts, significant disparities exist between formula-fed piglets and those receiving sow’s milk. Formula-fed piglets exhibit higher incidences of diarrhea, an elevated proportion of detrimental microorganisms in their intestines (Han et al., 2023; Zhang et al., 2022), and increased risks of necrotizing enterocolitis (NEC). Additionally, they show reduced levels of IgG and IL-2, despite faster weight gain rates (Berkhout Daniel et al., 2018; Cao et al., 2022). These findings raise the question of whether formula feeding can adequately replicate the effects of breastfeeding on gut microbiota structure and functionality (Berkhout Daniel et al., 2018; Saraf et al., 2017; Zhang et al., 2018b).

Recent advancements in metagenomic technologies have significantly enhanced our understanding of the effects of breastfeeding and formula feeding on gut microbiota. Studies have shown that breastfed infants exhibit higher gut microbial diversity, with an abundance of beneficial bacteria such as Bifidobacterium and Lactobacillus, which play critical roles in immune regulation and intestinal barrier function (Le Doare et al., 2018; Jang and Kim, 2022). Human milk oligosaccharides (HMOs), key components of breast milk, selectively promote the growth of Bifidobacterium while inhibiting the colonization of pathogenic bacteria. Additionally, immunoglobulins (e.g., IgA) and cytokines (e.g., IL-10) in breast milk modulate intestinal immune responses and enhance gut barrier function. In contrast, formula-fed infants tend to have lower gut microbial diversity and are more susceptible to the overgrowth of opportunistic pathogens such as *Clostridium difficile* and *Escherichia coli*, increasing the risk of intestinal inflammation and infections (Berkhout Daniel et al., 2018; Chong et al., 2022). Although probiotics (e.g., Lactobacillus and Bifidobacterium) are often added to formula milk to mimic the functions of breast milk, their efficacy is limited. Probiotics in formula milk may temporarily increase the abundance of certain beneficial bacteria, but their colonization capacity is weak, making it difficult to maintain long-term microbial balance (Le Doare et al., 2018; Jang and Kim, 2022). Furthermore, formula milk lacks natural immune components found in breast milk, such as antibodies (e.g., IgA), leukocytes, and bioactive molecules (e.g., lactoferrin and lysozyme), which are essential for antimicrobial and immunomodulatory functions. As a result, formula milk exhibits significant limitations in immune protection and gut microbiota regulation, potentially leading to incomplete immune system development and an increased risk of gut dysbiosis in infants.

The Bama pig, a native Chinese breed, has emerged as an ideal model for studying gut microbiota-host interactions due to the high similarity of its gut microbiota to that of humans (Azad et al., 2021). Studies have shown that the gut microbiota of Bama pigs holds significant value in research on metabolic diseases (e.g., obesity and diabetes) and immune-related disorders (e.g., inflammatory bowel disease) (Yang et al., 2015; Zeng et al., 2023). Additionally, the response of Bama pig gut microbiota to dietary interventions closely resembles that of humans, particularly in carbohydrate metabolism and the expression of antibiotic resistance genes (ARGs) (Zhu et al., 2022; Zhang et al., 2024). These characteristics make Bama pigs an excellent model for investigating the effects of different feeding methods on gut microbiota. In this study, we selected specific pathogen-free (SPF) Bama pigs as experimental subjects. SPF Bama pigs are raised in strictly isolated and sterile environments, free from a range of potential pathogens, ensuring the accuracy and reliability of experimental data (Lee et al., 2024; Yu et al., 2023). Additionally, the gut microbiota of SPF Bama pigs has lower abundance and exhibits higher sensitivity to minor environmental changes, facilitating clearer observations of the effects of feeding methods on gut microbiota. The well-defined genetic background and the unique features of the immune and digestive systems of SPF Bama pigs further enhance their suitability as a model for studying gut microbiota-host interactions (Yang et al., 2015; Zhang et al., 2024). This study aims to compare the effects of breastfeeding and formula feeding on the gut microbiota of SPF Bama pigs, focusing on differences in microbial diversity, metabolic functions, and ARGs, thereby providing a scientific basis for pig health management and infant feeding strategies.

## 2 Materials and methods

### 2.1 Animal trials and sampling

In this experiment, 20 healthy pregnant SPF-Bama sows of the same batch were utilized in the Shuanghe Experimental Base of the Bioengineering Institute of Chongqing Academy of Animal Science. To avoid the influence of genetic factors, 37 piglets were selected from 5 distinct lineages. Piglets from each lineage were randomly by draw the lot and evenly divided into two groups to ensure that there was no significant difference in the birth weight and body length of the piglets between the two groups (see Supplementary Table S1 and Supplementary Figure S1 for sample information). The breast milk group (Breast milk, BM, where sows and piglets lived together, raised in a single pen without manual intervention; and piglets had free access to feeding) and the formula group Formula Milk, FM, where piglets were kept in isolators and fed commercial suckling pig formula (the mixing ratio of formula milk and water was 1:6, and the composition and nutrition levels of the formula milk are shown in Supplementary Table S2). The feeding was conducted three times daily, with 300 mL per feeding during 0–7 days and 500 mL per feeding from 8 to 28 days. Throughout the entire experimental period, the management of piglets strictly adhered to the established protocols of the research center. The relative humidity within the pig housing was precisely regulated to remain within the range of 60%, while the room temperature was consistently maintained at 28°C. A 12-h light–dark cycle was implemented to simulate natural environmental conditions. Additionally, the use of any antibiotics or veterinary medications was strictly prohibited during the trial to ensure the purity and scientific rigor of the experiment. 31 piglets survived, with 16 in the BM group and 15 in the FM group. Fecal samples were collected from the piglets in both groups on days 7, 14, 21, and 28 of lactation (hereafter referred to as 7d, 14d, 21d, and 28d). Samples were collected at 10:00 am using anal swabs, which were then partitioned and placed in freezer storage tubes. Two additional fecal samples were collected from each piglet for further analysis. All samples were stored at −80°C.

### 2.2 DNA extraction and sequencing

Total fecal DNA was extracted by employing the E.ZastoolDNA Kit (OMEGA, USA) in accordance with the instructions for use. The extracted DNA samples were dispatched to Novogene Co., Ltd. for sequencing on the Illumina HiSeq Xten sequencing platform (Illumina, USA). The ultimate available sequencing data were as follows: BM: *n* = 64, 16 per time point; FM: *n* = 50, 12 per time point for 7d to 14d, and 13 per time point for 21d to 28d.

### 2.3 Metagenomic data analysis

Firstly, fastp (v0.23.2) (Chen et al., 2018) was employed to verify the sequencing quality provided by Novogene Co., Ltd. and eliminate low-quality reads, reads with trimmed lengths shorter than 50 were discarded. Subsequently, the reads were aligned with the pig host genome (Sus_scrofa.Sscrofa11.1) by means of bwa (v0.7.17-r1188) (Li and Durbin, 2009), and the unmapped sequences were extracted using samtools (v1.17) (Danecek et al., 2021) to obtain the gut microbiota genome after host removal. Megahit (v1.2.9) (Li et al., 2016) was utilized to assemble the genome of each sample, After comparison, the parameters were set as –min-count 2 –k-min 21 –k-max 141 –k-step 12 –min-contig-len 500, retaining contig sequences with lengths ≥500 bp. and Prodigal (v2.6.3) (Hyatt et al., 2010) was utilized for gene prediction. Then, all the samples were merged, and employed to construct a non-redundant (95% nucleotide identity) reference gene set via CD-HIT (v4.8.1) (Fu et al., 2012), Finally, Salmon (v1.10.1) (Patro et al., 2017) was used to align the non-redundant gene set with each sample’s de-host genome and calculate the relative abundance of genes.

### 2.4 Species and functional annotations

Kraken2 (Wood et al., 2019) is employed to annotate species for the assembled FASTA file. Seqkit converts the nucleotide sequences of the non-redundant gene set into amino acid sequences, and the amino acid sequences are uploaded to the KEGG online database^1^ for functional pathway annotation. db-CAN2 (v2.0.11) (Zhang et al., 2018a) is utilized for annotation of carbohydrate enzymes, and rgi (v6.0.3) (Alcock et al., 2023) is adopted for annotation of ARGs through the CARD database.

### 2.5 Statistical analysis

We predominantly employed statistical software R (v 4.3.2) for data statistical analysis. The *α* diversity of the overall gut microbiota was assessed using the Shannon index via the vegan (v 2.6–8) package, and the *β* diversity was computed using the phyloseq (v 1.46.0) package through the Bray-Curtis distance and Jaccard distance to generate a difference matrix and was visualized, concurrently, Permutational Multivariate Analysis of Variance (PERMANOVA) was utilized to confirm the significance. The inter-group differences of each gut microbiota were analyzed by means of the microeco package (v 1.9.1) for the effect size (LEfSe) analysis to determine the degree and significance of the differences (Segata et al., 2011); the 4 time points with the identical differences were selected and visualized by using pheatmap (v 1.0.12) to construct a heatmap. Ultimately, the correlation between gut microbiota and functions was evaluated using the psych package (v 2.4.3) through Spearman and Pearson correlation analysis, and Cytoscape is utilized to create the graph. The differences in dominant gut microbiota were visualized by means of the WeKemo Bioincloud online website,^2^ and the remainder were visualized using ggplot2.

## 3 Results and discussion

### 3.1 Differences in gut microbiota species and gene diversity between breastfeeding and formula feeding

**Figure 1:**
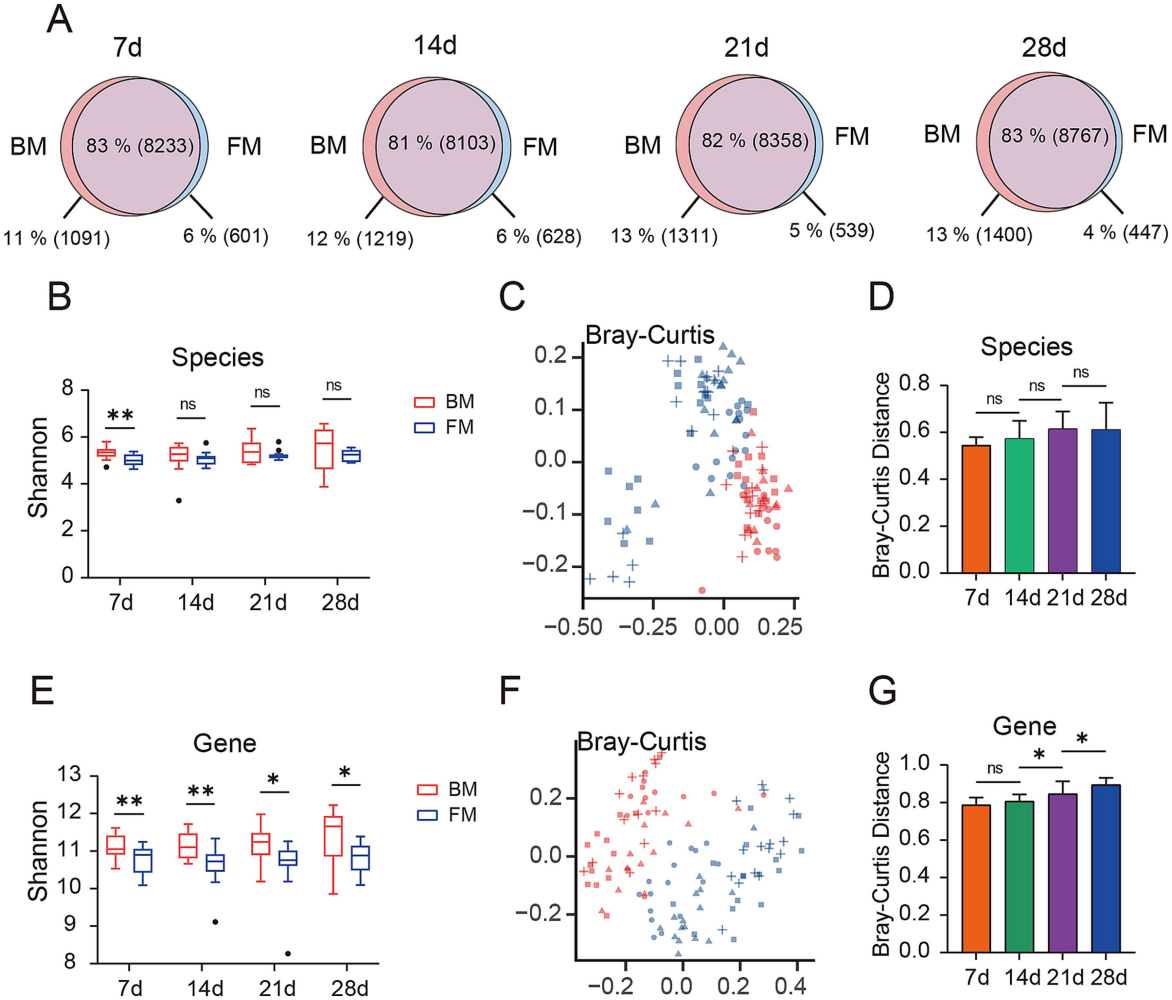
**(A)** Venn diagram of gut microbiota is presented, depicting the overlap (purple) and unique microbial species types between the BM group (red) and the FM group (blue) at four time points (7d, 14d, 21d, and 28d). The percentages represent the proportion of each section as a percentage of all microbial species observed at each time point for both groups, with the specific number of gut microbiota types represented in parentheses. **(B)** Alpha diversity of species between the two groups was compared by evaluating the Shannon index observed at the four time points. **(C)** Bray-Curtis distance matrix of species is presented on the principal coordinate analysis (PCoA) plot. **(D)** The difference in Bray-Curtis distance in terms of species between the BM group and the FM group at the same time points is shown. **(E)** The alpha diversity of genes between the two groups was compared by evaluating the Shannon index observed at the four time points. **(F)** The Bray-Curtis distance matrix of genes is presented on the principal coordinate analysis (PCoA) plot. **(G)** The difference in Bray-Curtis distance in terms of genes between the BM group and the FM group at the same time points is shown. * represents the *p*-value (*p ≤ 0.05, **p ≤ 0.01, ***p ≤ 0.001).

We employed Kraken2 to annotate species for 114 samples, these were annotated to 4 Kingdoms, 83 Phyla, 165 Classes, 314 Orders, 718 Families, 2,728 Genera, and 11,320 Species. We observed the common and endemic microbiota between the BM and FM groups at the species classification level (Figure 1A). Approximately 82% of the microbiota were shared between the BM and FM groups and remained approximately the same at all-time points. The proportion of microbiota unique to the BM group demonstrated an increasing trend over time (from 1,091 species accounting for 11% at 7d to 1,400 species accounting for 13% at 28d). Similarly, in the FM group, the proportion exhibited a decreasing trend over time (from 601 species accounting for 6% at 7d to 447 species accounting for 4% at 28d).

Prior to conducting the microbial diversity analysis, we initially employed the PERMANOVA test to confirm the microbial diversity among the four distinct time points, with a *p*-value of 0.001, thereby confirming the presence of significant differences between the different time points. Based on this finding, we proceeded to utilize the PERMANOVA test to affirm the influence of different experimental factors (diet, gender, and lineage) on microbial diversity, revealing that the diet factor exerted a significant impact on the gut microbiota at all four time points, while gender and lineage factors demonstrated no statistically significant differences (Supplementary Table S3).

We selected the Shannon index to assess the *α* diversity of the microbiota. The Shannon index of the BM group was higher than that of the FM group at each time point at the species level, and the uniformity of the BM group increased over time, reaching the highest level at 28d, while no significant change occurred in the FM group (Figure 1B). The visualization of the differences between groups through the application of the Bray-Curtis distance (Figure 1C) and Jaccard distance (Supplementary Figure S2A), along with the utilization of PERMANOVA to test for significant differences, can disclose a significant separation between the BM and FM groups (*p* < 0.001), and the disparity between the two groups augments over time (Figure 1D; Supplementary Figure S2B and Supplementary Table S4A). Under the same feeding mode, there were significant differences among all time points except 21d and 28d in the BM group (*p* < 0.001), and no significant differences between 21d and 28d in the BM group (*p* = 0.574). We also carried out alpha and beta diversity analysis at the gene level, and the results demonstrated a high consistency with the outcomes at the species level (there were significant differences between the BM and FM groups; aplha diversity increased with time in the BM group but did not change with time in the FM group) (Figures 1E–G; Supplementary Figures S2C,D and Supplementary Table S4B). The above results represented that with the passage of time, the diversity and structure of fecal microbiota would undergo changes under different feeding methods, and the diversity change in the BM group was significantly higher than that in the FM group. The diversity of the BM group at both the species and gene levels was significantly higher than that of the FM group. This result may be attributed to the richer microbial sources associated with the breastfeeding method. Breastfed piglets not only obtain nutrients from milk but are also exposed to microbes from the mother’s skin, feces, and the surrounding environment. These additional microbial sources likely contribute to the increased diversity of the gut microbiota (Jang and Kim, 2022). Furthermore, breast milk contains abundant human milk oligosaccharides (HMOs), immunoglobulins, and bioactive molecules. These components not only provide nutritional substrates for beneficial bacteria, such as Bifidobacterium and Lactobacillus, but also inhibit the colonization of pathogens, thereby maintaining the balance of the gut microbiota (Le Doare et al., 2018; Liu et al., 2019). In contrast, formula-fed piglets primarily rely on artificially formulated nutrition, lacking the complex immune components and microbial sources present in breast milk. This may lead to lower diversity in their gut microbiota (Chong et al., 2022).

### 3.2 Dominant microbiota and overall species differences

**Figure 2:**
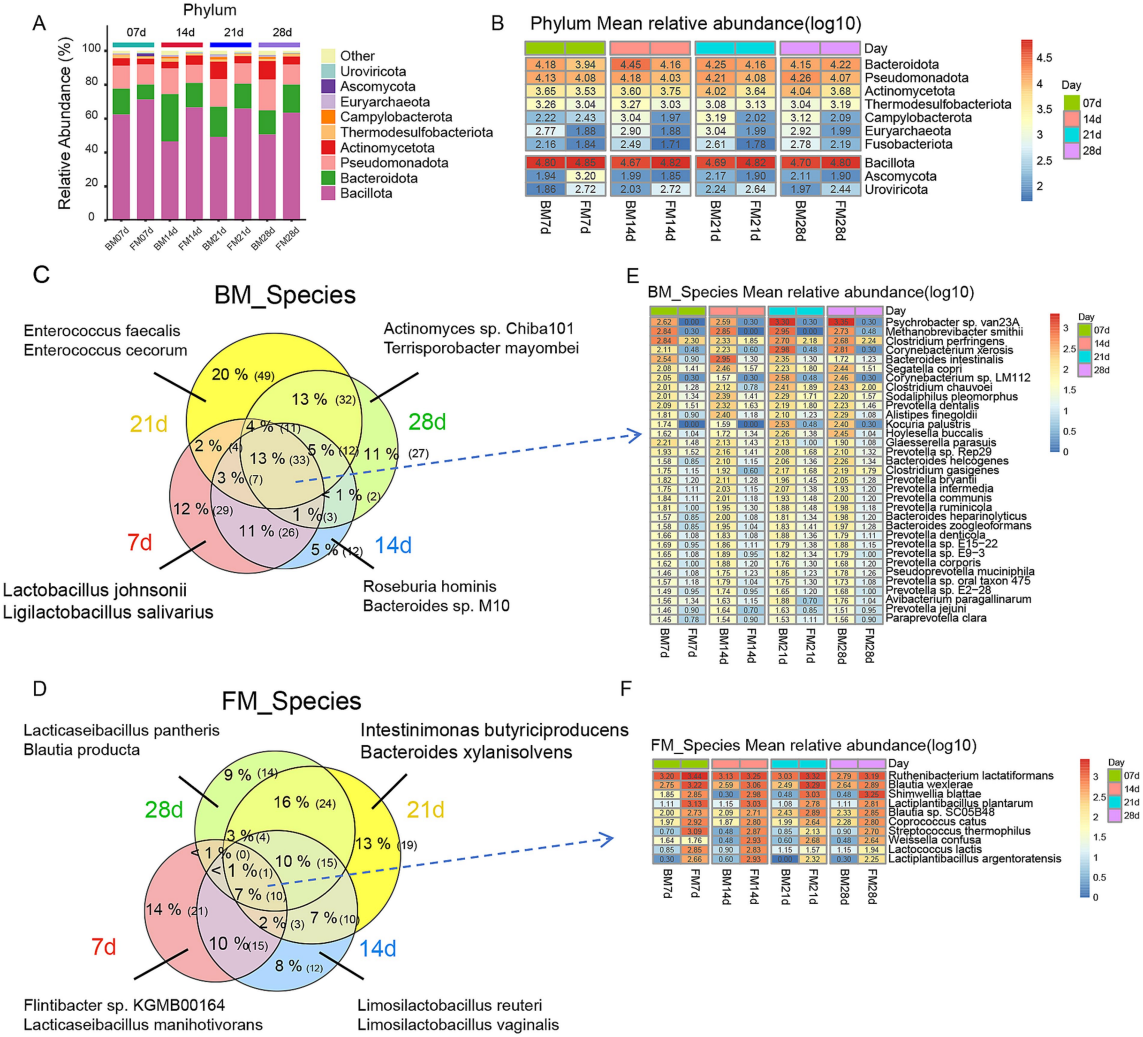
**(A)** Bar plot is presented to represent the relative abundance of dominant species at four time points within the BM and FM groups at the phylum level. The vertical axis represents the proportion of species in the sample. Different colored columns signify different species, and the length of the columns represents the proportion of the species. **(B)** Heatmap is presented to illustrate the significant differences in gut microbiota between the two groups at more than three time points at the phylum level. Above the gap, the microbial phyla in the BM group are significantly higher than those in the FM group; below the gap, the microbial phyla in the FM group are significantly higher than those in the BM group. **(C,D)** Venn diagram of differential species. The number of species with overlapping and unique gut microbiota that were observed in the two groups at 7d (red), 14d (blue), 21d (yellow), and 28d (green). Percentages represent the proportion of each section constituting all the differential microbial species in the group, and the numbers in parentheses represent the specific number of gut microbiota. **(E)** Heatmap is presented to represent the gut microbiota that were significantly higher in the BM group at all four time points at the species level. The heatmap is sorted by the average relative abundance in log10 scale. The numerical values on the color scale represent the relative abundance after being rounded to the nearest 10,000 and then log10 transformed. **(F)** Heatmap is presented to represent the gut microbiota that were significantly higher in the FM group at all four time points at the species level. The heatmap is sorted by the average relative abundance in log10 scale. The numerical values on the color scale represent the relative abundance after being rounded to the nearest 10,000 and then log10 transformed.

Next, we, respectively, analyzed the dominant microbiota at the Phylum level and Species level using the group mean values of relative abundance. The visualization of the top 15 phyla by accumulation map demonstrated that the dominant phyla of the two groups were Bacillota, Bacteroidota, Pseudomonadota, and Actinomycetota in the fecal microbiota of lactating piglets (Figure 2A). Among them, the relative abundance of Bacillota was significantly higher than that of the BM group at four time points (*T*-test, *p* < 0.005), significantly decreased at 14d in the BM group (*T*-test, *p* < 0.0001), and then increased over time, but not significantly, while in the FM group it decreased over time, but not significantly. However, the relative abundance of Bacteroidota in the BM group was significantly higher than that in the FM group at 7d - 14d (*T*-test, *p* < 0.01), and there was no significant difference between the two groups at 21d - 28d (Supplementary Table S5). At the Species level, *Escherichia coli* was the most abundant strain in both groups, and *Flavonifractor plautii*, the second dominant microbiota, had the maximum enrichment at 7d and an apparent reduction at 14d in the BM group (*T*-test, *p* < 0.0001). *Bacteroides fragilis* increased significantly at 14d in the BM group (*T*-test, *p* < 0.0001) and was relatively low in the remaining time points (Supplementary Figure S3A and Supplementary Table S6A). In the FM group, *Enterocloster clostridioformis* and *Enterocloster bolteae* were the next dominant, and there was no significant change in each time point (Supplementary Figure S3B and Supplementary Table S6B). In the BM group, *Flavonifractor plautii* and *Bacteroides fragilis* are key microbiota supporting gut health and growth in piglets. *F. plautii* degrades flavonoids, aiding microbiota balance and potentially reducing obesity-related inflammation, which may improve feed efficiency (Tourlousse et al., 2020; Mikami et al., 2020). *B. fragilis* secretes antimicrobial proteins (BfUbb) to enhance competition and regulates gut immunity through fucosylation, crucial for disease resistance (Jiang et al., 2024; Ramakrishna et al., 2019; Lei et al., 2025). In the FM group, *Bacteroides thetaiotaomicron* and Limosilactobacillus reuteri play vital roles. *B. thetaiotaomicron* degrades polysaccharides, producing SCFAs that strengthen the intestinal barrier and nutrient absorption (Krautkramer et al., 2021). *L. reuteri*, combined with GOS, reduces inflammation and enhances gut barrier function through SCFA production, supporting growth and feed utilization (Abuqwider et al., 2022; De Bruyn et al., 2024).

Furthermore, the difference analysis of Phylum and Species levels between the two groups was conducted by LEfSe (LDA > 2, *p* < 0.05), and the species with significant differences between the two groups were selected at each time point. Subsequently, a heat map was generated based on the average relative abundance log10. At the Phylum level, 10 species exhibited significant differences at more than 3 time points between the two groups (Figure 2B). Bacillota was significantly higher in the FM group than in the BM group at all four time points. The average proportion of Bacillota in the FM group was 71.28% at 7d and 62.43% in the BM group (*p* = 0.0046), which gradually decreased over time. The average proportion of Bacillota in the FM group was 63.51 and 50.56% in the BM group at 28d (*p* = 0.00573). Conversely, Pseudomonadota in the BM group was significantly higher than that in the FM group in all the 4 time points. The average proportion in the BM group was 13.5 and 11.94% in the FM group at 7d (*p* = 0.046), 18.29% in the BM group and 11.86% in the FM group at 28d (*p* = 0.0057). In the BM group, the proportion presented a gradual upward trend (not significant). The average proportion of Bacteroidota in the BM group at 7d and 14d was higher than that of the FM group (7d: *p* = 0.0062, 14d: *p* < 0.001), but then decreased, and there was no significant difference between the two groups at 21d and 28d. There was no significant difference in the ratio of Actinomycetota at 7d, but the difference increased at 21d, with 10.76% in the BM group and 4.38% in the FM group (*p* = 0.023), and 11.19% in the BM group and 4.76% in the FM group at 28d (*p* < 0.001). Additionally, Campylobacterota had no significant difference at 7d and was significantly higher in the BM group than in the FM group from 14d to 28d. Euryarchaeota in the BM group was significantly higher than that in the FM group at 4 time points, and Uroviricota in the FM group was significantly higher than that in the BM group at 4 time points.

At the Species level, we also employed the LEfSe method (LDA > 2, *p* < 0.05) to conduct an in-depth analysis of gut microbiota differences at different time points. Since the microbiota differences between the BM group and the FM group represented distinct characteristics at each time point, we utilized Venn diagram analysis to identify the common and unique different microbiota at each time point. At all-time points, a total of 33 species were significantly more abundant in the BM group than in the FM group, and these species presented this trend at all four time points (Figures 2C,E). Among them, Psychrobacter sp. van23A, *Methanobrevibacter smithii*, and *Corynebacterium xerosis* are the most dominant bacterium groups. However, only 10 of them are enriched in the FM group, with Lactobacillaceae (3 species) and Lachnospiraceae (three species) being the most prevalent (Figures 2D,F). Among the time-specific differential microbiota, the time point at which the different species were most enriched was 21d (BM: 60%, FM: 55%), and a considerable proportion of these species only manifested differences at 21d (BM: 20%, FM: 13%). At 21d, the BM group showed higher abundance of *Enterococcus faecalis* and *Enterococcus cecorum*. *E. faecalis*, a common gut bacterium, aids in microbial balance, inhibits harmful bacteria, and supports immune development (Boeder et al., 2024). *E. cecorum* colonizes well and modulates bile acid metabolism via bile salt hydrolase (Wu et al., 2022a). Conversely, the FM group had more *Bacteroides xylanisolvens* and *Intestinimonas butyriciproducens*. *B. xylanisolvens* degrades complex carbs, producing SCFAs like acetate and butyrate, which support gut barrier function and metabolism (Fu et al., 2023). *I. butyriciproducens* converts fructoselysine into butyrate and acetate, improving gut health and metabolic functions (Rampanelli et al., 2025). At 7d, it was discovered that more species were relatively enriched and their relative abundance was higher in the BM group, but the relative abundance of more than 1/3 species in the FM group gradually decreased from 14d to 28d, and was significantly higher in the FM group than in the BM group at the later stage. The relative abundance of *Lactobacillus johnsonii*, *Limosilactobacillus mucosae*, and *Ligilactobacillus salivarius* in the Lactobacillaceae family was higher. The relative abundance of FM-enriched species was high from 21d to 28d, and the species belonging to Lactobacillaceae, Lachnospiraceae, and Oscillospiraceae were the largest, which exceeded more than half of the group. In addition, other species in the FM group from 21d to 28d included *Thomasclavelia [Clostridium] innocuum*, *Hungatella hathewayi*, and *Parabacteroides goldsteinii*, *Phascolarctobacterium faecium*, etc., all of which have been reported to participate in the process of carbohydrate decomposition and SCFAs generation (Figures 2C,D; Supplementary Figure S4) (Cui et al., 2022; Del Dot et al., 1993; Kaur et al., 2014; Bhattacharjee et al., 2022). In conclusion, during lactation, the fecal microbiota had the greatest difference between groups at around 21d, and the fecal microbiota involved in carbohydrate decomposition and SCFAs production would gradually enrich in the FM group at around 21d. SCFAs is the preferred energy metabolic source of intestinal epithelial cells, which is mainly produced by oligosaccharides in breast milk through intestinal microbial decomposition (Inoue and Tsukahara, 2021; Li et al., 2019).

### 3.3 Functional pathways

**Figure 3:**
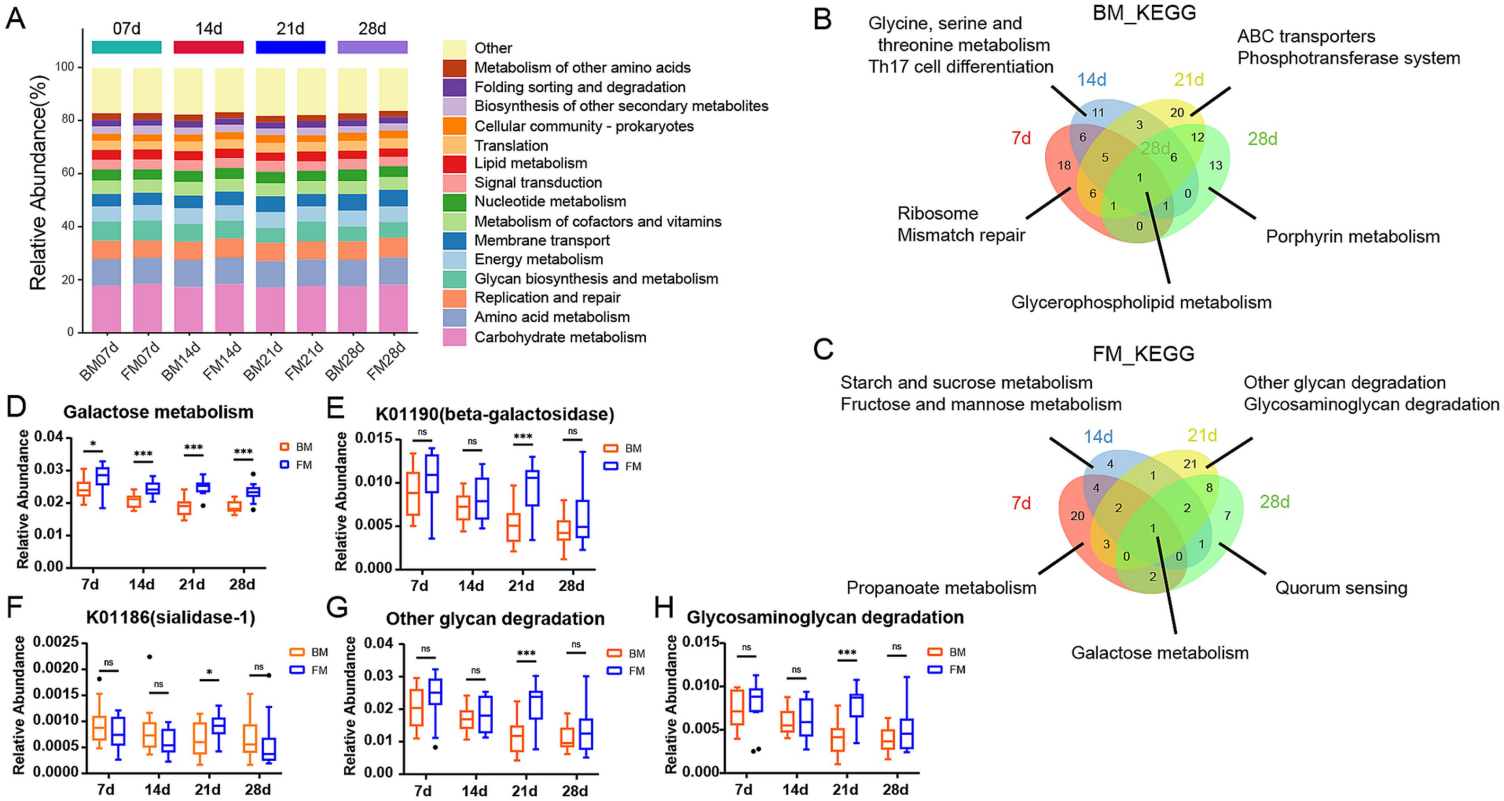
**(A)** Stacked bar chart is presented to represent the relative abundance of the dominant functions at the 4 time points within the KEGG B-level classification of the BM group and the FM group. The vertical axis represents the proportion of functions in the sample. Different colored columns signify different B-level classification functions, and the length of the columns represents the proportion of species. **(B,C)** Venn diagram of differences KEGG. The number of overlapping and unique KEGG pathway was observed in the two groups at 7d (red), 14d (blue), 21d (yellow), and 28d (green). The numbers in parentheses represent the KEGG pathway in each overlapping or unique group. **(D)** Boxplot is presented to represent the relative abundance of Galactose Metabolism in the two groups at the 4 time points. **(E,F)** Boxplot presenting the relative abundance of K01190 and K01186 at four time points. **(G,H)** Boxplot presenting the relative abundance of other glycan degradation and Glycosaminoglycan degradation at four time points. *represents the *p*-value (*p ≤ 0.05, **p ≤ 0.01, ***p ≤ 0.001).

To explore the functional differences of microbiota in different feeding styles during various time points, we carried out KEGG Orthology annotation on fecal microbial function in 4 time points and obtained a total of 14,066 KO. The function with the highest relative abundance was Carbohydrate metabolism, accounting for approximately 18% at different time points in both groups, followed by Amino acid metabolism (Figure 3A). This is primarily due to the high demand for energy and protein during the rapid growth and development of piglets. Carbohydrates, particularly lactose, serve as the main energy source, meeting the high energy requirements of piglets (Forsgård, 2019). Simultaneously, amino acid metabolism is crucial for supporting the growth of muscles, bones, and other tissues in piglets. Proteins in breast milk and formula are broken down into amino acids to fulfill these needs (Inoue and Tsukahara, 2021). Additionally, gut microbiota play a key role in the decomposition and utilization of carbohydrates and amino acids. Oligosaccharides, lactose, and proteins provide abundant substrates for gut microbiota, promoting the growth of beneficial bacteria and further enhancing the activity of related metabolic pathways (Le Doare et al., 2018). These factors collectively contribute to the prominent representation of carbohydrate and amino acid metabolism pathways under both feeding styles. We employed LEfSe to explore the differences between the BM and FM groups at four distinct time points and discovered that there were exceedingly few shared differential functions across all time points. The sole shared differential functions were Glycerophospholipid metabolism in the BM group and Galactose metabolism in the FM group. There were more time-specific functional groups at time points 7d and 28d (Figures 3B,C).

In the FM group, the functions were significantly higher than those in the BM group. Except for Galactose metabolism, 7–14d mainly focused on Starch and sucrose metabolism. 21–28d was mainly Amino sugar and nucleotide sugar metabolism. At 21d, other glycan degradation, glycosaminoglycan degradation, glycosphingolipid biosynthesis—globo and isoglobo series in the FM group and Various types of N - glycan biosynthesis in the FM group were significantly higher than those in the BM group, all of which were functions related to glycan biosynthesis and metabolism. Alanine, aspartate and glutamate metabolism and other Amino acid metabolism functions were also significantly enriched in the FM group at 28d (Figure 3C; Supplementary Figure S5). The functional differences in the gut microbiota at different time points may be related to their nutritional composition and adaptive regulation of the microbiota.

In the BM group, pathways such as aminoacyl-tRNA biosynthesis, mismatch repair, purine metabolism, and peptidoglycan biosynthesis were significantly higher than those in the FM group at 7d. These pathways are mainly involved in various functions related to bacterial reproduction, including amino acid and protein synthesis, gene replication, and cell wall construction (Polycarpo et al., 2004; Li, 2008). From 14d to 28d, Flagellar assembly and Bacterial chemotaxis were the most significantly enriched functions of the BM group, all of which were Cell motility functions. In addition, Membrane transport functions such as ABC transporters and Phosphotransferase system (PTS) were significantly enriched in the BM group at 21d (Figure 3B; Supplementary Figure S5).

It can be observed that piglets in the BM group fed by sows have a greater advantage in bacterial reproduction, which might be related to the greater microbial diversity, while piglets in the FM group fed by artificial formula have a greater advantage in metabolism. However, the metabolic pattern differed at different time points.

Given that Galactose Metabolism in the FM group is significantly higher than that in the BM group at all four time points, a box diagram of the Galactose Metabolism pathway shows that the relative abundance of the FM group decreases as a whole, but increases at 21d. As a result, it is significantly higher than that in BM group at 21d (Figure 3D). KO genes in the Galactose metabolism pathway include beta-galactosidase (K01190), beta-galactosidase (K12308), alpha-glucosidase (K01187), alpha-galactosidase (K07407) and aldose 1-epimerase (K01785), which are mainly involved in the decomposition of lactose to galactose and glucose, and further transformation into other monosaccharides. The average relative abundance of beta-galactosidase (K01190) is more than 10 times that of other KO genes, and it plays the most important role in galactose metabolism. Beta-galactosidase is mainly responsible for catalyzing the hydrolysis of lactose into galactose and glucose, two monosaccharides that are more easily absorbed and utilized by the intestine. Beta-galactosidase (K01190) was consistently higher in the FM group than in the BM group, but was significantly elevated and significantly higher than in the BM group at 21d (Figures 3E,F).

In addition, there are significant differences in several Glycan biosynthesis and metabolism pathways such as Other glycan degradation and Glycosaminoglycan degradation at 21d. We also used boxplot to show that the relative abundance of metabolism in both groups showed a similar trend to that of Galactose Metabolism (Figures 3G,H). The KO genes in the Other glycan degradation pathway include sialidase-1 (K01186), beta-galactosidase (K01190), alpha-L-fucosidase (K01206), alpha-L-fucosidase 2 (K15923), non-lysosomal glucosylceramidase (K17108), Among them, beta-galactosidase (K01190) is still the most important KO gene affecting the relative abundance changes of Other glycan degradation pathways, but the enzyme that directly metabolizes N-Acetylneuraminic Acid (Neu5Acα2) within the metabolic pathway is sialidase-1, which belongs to sialic acid and is an important nine-carbon sugar derivative in breast milk oligosaccharides. Sialidase-1 in FM group was lower than that in BM group at 7d, 14d and 28d, but it was significantly increased and significantly higher than that in BM group at 21d, which reflected the changes of oligosaccharide metabolism by microbiota to a certain extent.

In our study, the galactose metabolic pathway and *β*-galactosidase (K01190) of KEGG were significantly elevated in the four time points within the FM group. This might be attributed to the fact that the formula milk we employed was predominantly composed of cow milk powder derived from cow milk. The lactose content in cow milk was approximately twice that in pig milk, and the higher lactose concentration exceeded the digestive capacity of piglets. The excess lactose entered the colon and provided a fermentation substrate for intestinal microbes (Wu et al., 2022b). Lactose is a significant carbohydrate in breast milk and is synthesized in the mammary gland. It is mainly catalyzed in the small intestine by Lactase-phlorizin hydrolase (LPH) into glucose and galactose, then it is hydrolyzed by galactosidase and absorbed (Forsgård, 2019). The content of oligosaccharides in breast milk is much lower than that of lactose, yet it is an essential component of breast milk. Since human and pig intestines lack enzymes to decompose oligosaccharides, the majority of oligosaccharides cannot be directly digested, thus intestinal microbes are indispensable contributors (Inoue and Tsukahara, 2021; Li et al., 2019). Gut microbiota decompose oligosaccharides into simple sugar molecules by secreting specific enzymes, enabling them to be absorbed and utilized by the host. For instance, sialidase-1 (K01186) can break down sialic acid in oligosaccharides, releasing monosaccharides that can be utilized by the host while providing an energy source for the microbiota themselves. Additionally, the microbial metabolism of oligosaccharides promotes the proliferation of beneficial bacteria, which can inhibit the growth of harmful bacteria, maintain gut microecological balance, and thus positively impact the host’s intestinal health and immune function (Muñoz-Provencio and Yebra, 2023). This microbial-mediated oligosaccharide metabolism not only supports the host’s nutritional needs but also exerts profound effects on the structure and function of the gut microbiota. Additionally, we also observed that glycan-related metabolic pathways and galactose metabolic pathways were significantly augmented in the FM group at 21d and were significantly higher than those in the BM group. The β-galactosidase (K01190) plays a crucial role in Other glycan degradation pathways in addition to its major functions in galactose metabolism. It is a key gene in the conversion of N-Acetylneuraminic Acid to ceramide. Furthermore, Other glycan degradation factors include sialidase-1 (K01186), alpha-L-fucosidase (K01206), alpha-L-fucosidase (K15923), Glycosaminoglycan degradation is mainly associated with hyaluronoglucosaminidase (K01197), alpha-N-acetylglucosaminidase (K01205), hexosaminidase (K12373). All of these KO genes exhibited a significant upward trend at 21d of the FM group. These results all represented that the microbiota related to glycan metabolism would be more concentrated in the FM group at 21d, which was consistent with the previous results of microbiota changes. This enrichment phenomenon is likely closely related to the compositional characteristics of the formula milk and the developmental stage of the host’s intestine. The higher lactose and protein content in the formula milk provides a rich fermentation substrate for the microbiota, especially at 21d when the gut microbiota of piglets gradually matures and becomes more efficient at utilizing these complex nutrients. At this stage, microorganisms associated with glycan metabolism undergo selective enrichment, enhancing their ability to metabolize lactose and oligosaccharides, thereby exhibiting higher abundance and activity in the FM group. This dynamic change reflects the adaptability of the gut microbiota to different nutritional environments and also highlights the unique role of formula milk in shaping the functional profile of the gut microbiota.

### 3.4 CAZymes

**Figure 4:**
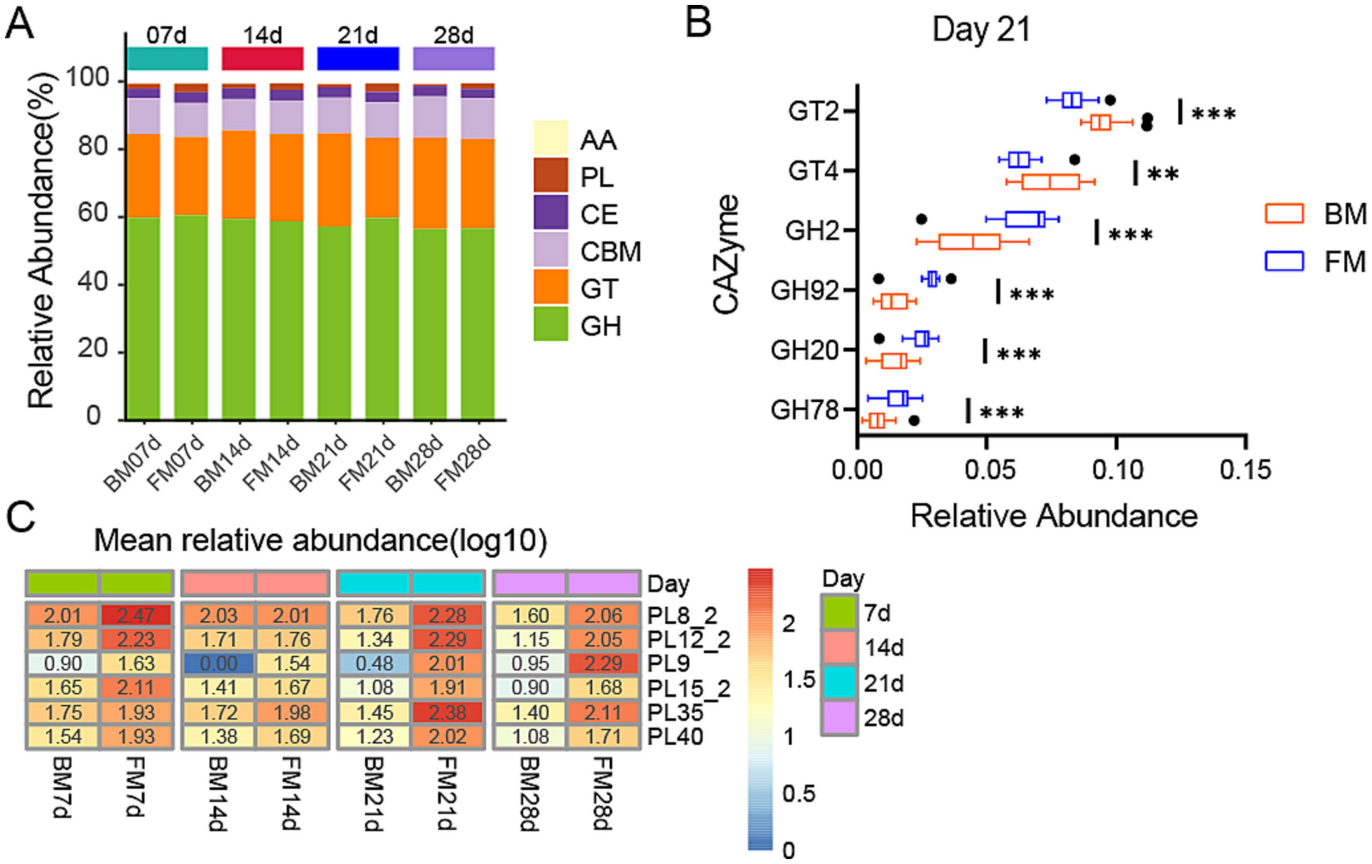
**(A)** Heatmap represents the relative abundance of six CAZyme families in two groups at four time points. The vertical axis represents the proportion of carbohydrate-active enzyme families within the sample. The columns of different colors represents distinct classes of carbohydrate-active enzyme families, and the length of the columns represents the proportion of carbohydrate-active enzymes. **(B)** Boxplot is presented for the LEfSe analysis of GHs and GTs in two groups at 21d, with LDA > 3.5 and *p* < 0.05. The horizontal axis represents the relative abundance, and the vertical axis represents the names of enzyme families. GT and GH are sorted in a descending order of LDA from the largest to the smallest. **(C)** Heatmap of differential PLs families at four time points in two groups. The heatmap is sorted by the average relative abundance on a log10 scale. The numerical values on the color scale are the outcome of rounding the relative abundance X 10000 to the nearest integer and then performing log10 processing on the result. * represents the *p*-value (*p ≤ 0.05, **p ≤ 0.01, ***p ≤ 0.001).

As represented by KEGG functional annotations, significant differences exist in Carbohydrate metabolism. Through the CAZy Database (Carbohydrate-Active enZYmes Database), we annotated carbohydrate-active enzymes (CAZymes) for further analysis of carbohydrate-related functions. A total of 484 CAZymes were obtained, which belong to 6 families, including 272 Glycoside Hydrolases (GH), 70 Carbohydrate Binding modules (CBM), 70 Glycosyl Transferases (GT, glycosyltransferase), 50 Polysaccharide Lyases (PL, polysaccharide lyase), 17 Carbohydrate Esterases (CE, carbohydrate esterase), and 8 Auxiliary Activities (AA, oxidoreductase). GHs are capable of breaking down complex carbohydrates into simple sugar molecules, providing an energy source for gut microbiota while enhancing the host’s absorption of nutrients. CBMs assist enzymes in better binding to substrates, thereby improving metabolic efficiency. GTs are involved in the synthesis and repair of cell walls, playing a crucial role in maintaining the structural integrity of gut microbiota. PLs and CEs participate in the degradation and de-esterification of polysaccharides, respectively, facilitating the release of nutrients that can be utilized by the host. The activity and diversity of these enzymes directly influence the intestinal health, nutrient absorption, and immune responses of piglets, thereby profoundly impacting their growth performance and disease resistance. GHs was the category with the highest relative abundance, accounting for approximately 60% in both groups during the four time points, and in the FM group, it was significantly higher than the BM group at 21d (*p* = 0.0023), while there was no significant difference in other time points. GTs ranked second, accounting for about 23–27%, and in the BM group was significantly higher than the FM group at 21d (*p* = 0.0002), while there was no significant difference in other time points. Additionally, PLs were significantly enriched in the FM group at 7d, 21d, and 28d, while no significant differences were observed at 14d (Figure 4A).

To determine which enzymes among these families exert the most significant impact on the dissimilarities between the two groups, we conducted a more in-depth analysis of the distinctions in PLs between the two groups at three time points (7d, 21d, 28d) and the variances in GHs and GTs between the two groups at 21d using the LEfSe method. At 21d (LDA > 3.5, *p* < 0.05), the GT family exerting the most significant impact on BM was primarily GT2 and GT4, which were conspicuously enriched in the BM group. In the FM group, the GH family was markedly enriched, with the most pronounced impact encompassing GH2, GH92, GH20, and GH78 (Figure 4B). Among the PLs that were conspicuously enriched in the FM group (LDA > 2.0, *p* < 0.05), PL8_2, PL12_2, PL15_2, PL9, and PL40 were consistently higher in the FM group than in the BM group, while PL35 was merely significantly higher in the FM group at 21d and 28d (Figure 4C).

GTs are involved in various biological processes in animals, encompassing cell signaling, cell adhesion, receptor activation, signal transduction, and endocytosis (Moremen and Haltiwanger, 2019). GT2 and GT4 can synthesize cellulose and chitin, along with other basic structural components, and play a crucial role in the formation and protection of cell membranes and cell walls (Alshareef, 2024; Gallicchio et al., 2019), which might promote the proliferation of microbiota. In the previous results, the Membrane Transport pathway of KEGG was most enriched in the BM group at 21d, which could be related to the activity of GT2 and GT4.

GHs play a key role in the decomposition of intestinal carbohydrates. These enzymes facilitate microorganisms to break down host glycans (Henrissat, 1991). For instance, the GH2 family contains *β*-galactosidase, which can participate in other glycan degradation pathways. β-glucuronidases from the GH2 family are also implicated in glucosylation products involved in drug metabolism (Wardman et al., 2022). The mannosyl-oligosaccharide, 1,2-*α*-mannosidase of the GH92 family is involved in the maturation process of n-glycan (Tabas and Kornfeld, 1979). GH20 can catalyze the breakdown of HMOs and mucin, and also influence the glycosylation of antibiotics to enhance biological resistance (Wardman et al., 2022). The enrichment results of these GHs families in the 21d FM group are consistent with the results of glycan-related metabolic pathways in KEGG.

PLs are involved in the degradation of complex polysaccharides in intestinal microorganisms (Lombard et al., 2010). The levels of GHs and PLs in the FM group were significantly higher than those in the BM group, indicating that carbohydrate and polysaccharide degradation activities were more vigorous in the gut microbiota of piglets in the FM group. And these PL families mostly comprise chondroitin lyase (PL35, PL8_2) and heparin lyase/heparin sulfate lyase/heparan sulfate lyase (PL12_2, PL15_2, PL8_2), which can act on glycosaminoglycans (GAG). Glycosaminoglycan is a type of biopolymer that plays a key role in various biological metabolic processes such as cell signaling, tissue development, cell apoptosis, immune regulation, and growth factor activity. They exist primarily in a free form in the colon and maintain host health by regulating the colonization and proliferation of the gut microbiota. The amount of glycosaminoglycan in milk is approximately 60.2 mg/L, of which galactosaminoglycan is 60.1% (Chondroitin sulfate (CS) 21.4%, dermal sulfate (DS) 39.7%), heparin (Hep) 34.4%, and hyaluronic acid (HA) 4.5% (Coppa et al., 2010; Wang and Chi, 2022). These GAGs can all be cleaved by the above-mentioned PL families, suggesting that the microbiota associated with glycosaminoglycan degradation might be more active in the FM group.

### 3.5 Gut microbiota associated with polysaccharide metabolism

**Figure 5:**
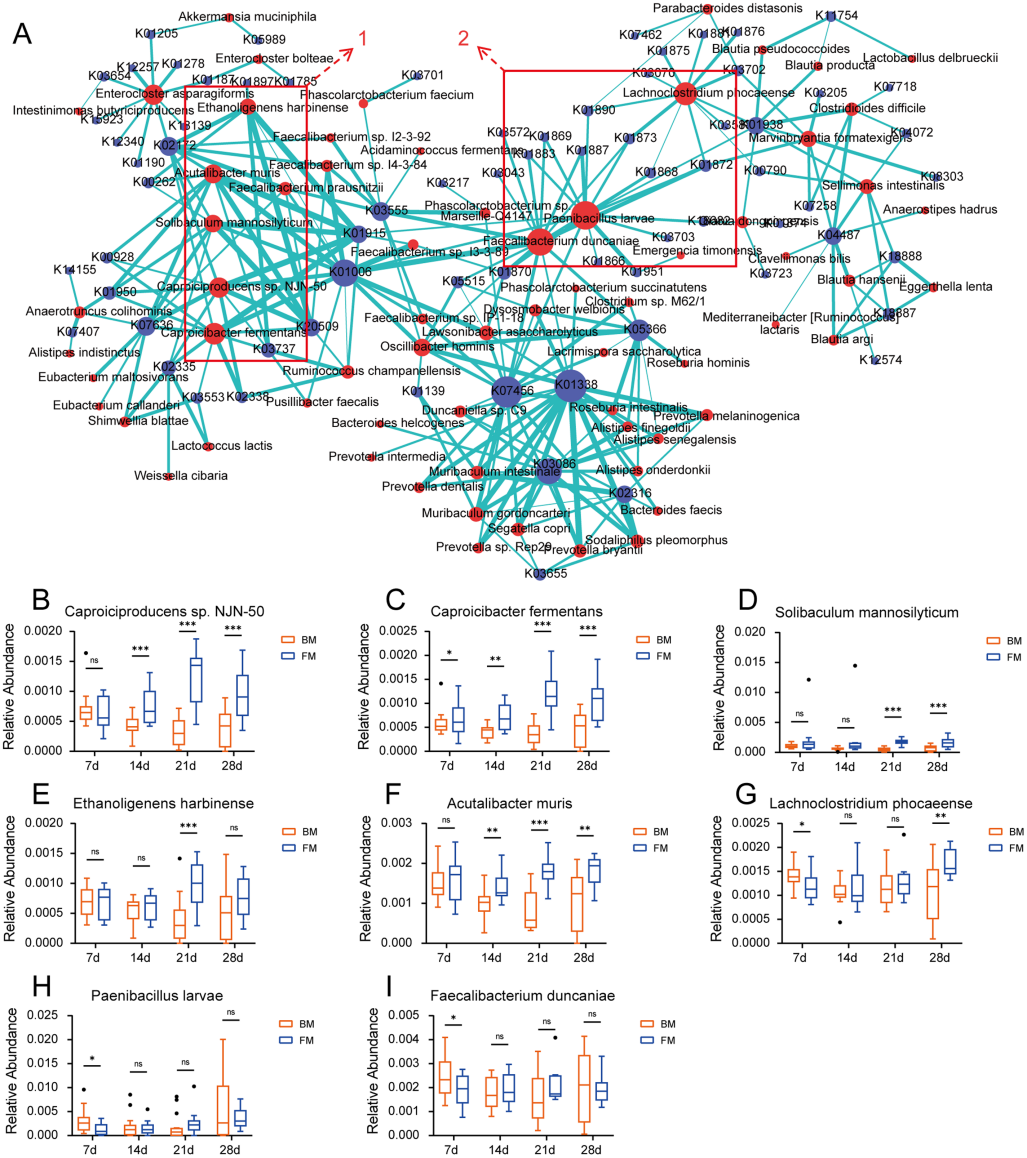
**(A)** Network diagram of the correlation between species and KO genes. The relationship coefficient *r* > 0.5, edges = green. Species = red, KO = blue. **(B–I)**. Boxplots of the relative abundance of “hub species.” * represents the *p*-value (*p ≤ 0.05, **p ≤ 0.01, ***p ≤ 0.001).

In order to validate the relationship between microbiota and various enzymes, we performed Spearman correlation analyses between species with a relative abundance >0.001 and KO genes, as well as CAZymes, and those with a correlation coefficient *r* > 0.5 were considered to have a high positive correlation (*p* < 0.001). Consequently, the elements with *r* ≥ 0.5 were selected, and the correlation between species and KO gene as well as species and CAZymes was presented on the network map using Cytoscape. As can be observed from Figure 5A, the species clusters set 1 is defined based on the most highly correlated and densely connected group of five species in the correlation network diagram. Within the KO-associated, this cluster encompasses *Caproiciproducens* sp. NJN-50, *Caproicibacter fermentans, Solibaculum mannosilyticum*, *Ethanoligenens harbinense*, and *Acutalibacter muris*, all of which belong to the family Oscillospiraceae of the Phylum Bacillota. These species exhibited a significantly higher relative abundance in the FM group when compared to the BM group from 14d to 28d (*Ethanoligenens harbinense* was significantly higher than that in the BM group at 21d - 28d). The related KO genes are more involved in the energy metabolism cycle (such as the Citrate cycle, Pyruvate metabolism, Glycolysis/Gluconeogenesis, and other functional pathways), including Pyruvate-Ferredoxin/flavodoxin oxidoreductase (K03737), carboxybiotin decarboxylase (K20509), pyruvate, orthophosphate dikinase (K01006), etc. These species may gradually colonize the piglet intestine and provide more energy to the host after 14d (Figures 5B–F). Another species clusters set 2 focuses on *Paenibacillus larvae*, *Faecalibacterium duncaniae, Lachnoclostridium phocaeense*, mainly related to Aminoacyl-tRNA biosynthesis. Including alanyl-tRNA synthetase (K01872), threonyl-tRNA synthetase (K01868), valyl-tRNA synthetase (K01873), arginyl-tRNA synthetase (K01887), cysteinyl-tRNA synthetase (K01883), and other 12 Aminoacyl-tRNA synthetase enzymes. These enzymes mainly provide raw materials for protein synthesis, ensure the accurate transmission and expression of genetic information, and affect the growth and differentiation of cells. The three species showed no significant difference between the two groups for most of the time; only *Paenibacillus larvae* and Lachnoclostridium phocaeense were significantly higher in the BM group than in the FM group at 7d (Figures 5G–I).

The network diagram of species related to the CAZymes is presented in Supplementary Figure S6. We discovered that the clustered species with a high positive correlation to the CAZymes in the network diagram mainly comprised *Caproiciproducens* sp. NJN-50, *Ethanoligenens harbinense*, *Solibaculum mannosilyticum*, etc., which exhibits a certain degree of overlapped with the species of group 1 in the KO association cluster. Simultaneously, we found that the CAZymse were mainly associated with the PL families in this cluster. These PLs all manifested in the previous results that their relative abundance was significantly higher in the FM group than in the BM group. We further performed Spearman and Pearson correlation analyses on the species that were significantly higher in the FM group than in the BM group and the CAZymes related to glycan metabolism in the previous results. We discovered that four species in the CAZymes had a highly positive correlation with the PL35 enzyme family (chondroitin lyase/chondroitinase) in both the Spearman and Pearson correlation coefficients with a correlation coefficient r > 0.5. These included *Acutalibacter muris*, *Ethanoligenens harbinense*, *Caproiciproducens* sp. NJN-50, and Caproicibacter fermentans. All of these species are in group 1, which is concentrated in association with KO (Supplementary Table S7).

The PL35 family encompasses Chondroitin lyase, Hyaluronate lyase, and Heparin-sulfate lyase, the substrates of which are all components of glycosaminoglycans (including chondroitin, hyaluronic acid, and heparin sulfate, among others). These glycosaminoglycan components are alternately linked by GalNAc or GlcNAc to uronic acid via glucoside linkages. The PL35 family degrades these glycosaminoglycan components by cleaving the glucoside linkages (Wang et al., 2017; Lu et al., 2024). Although *Acutalibacter muris*, *Ethanoligenens harbinense*, *Caproiciproducens* sp. NJN-50, and *Caproicibacter fermentans* were not reported to be directly associated with glycosaminoglycan metabolism, all four species belong to the family Oscillospiraceae, which has been reported to degrade mucin (Raimondi et al., 2021). There are also GalNAc and GlcNAc glycogroups in mucins; thus, the mechanism of mucin degradation by microbiota may be related to the degradation of glycosaminoglycans (Malaker et al., 2022). However, these mechanisms need to be verified through further experiments. The highly correlated performance of these microbiota simultaneously in the energy metabolic cycle suggests that they may play a crucial role in regulating the metabolic health and energy balance of their hosts. These energy metabolic cycle processes generate SCFAs, lactic acid, acetic acid, etc., which provide the host with additional energy and promote host growth (Sowah et al., 2019).

### 3.6 ARGs

Finally, we confirmed the disparity of ARGs in piglets’ feces during distinct time points of formula feeding. We annotated ARGs using the CARD database, and a total of 331 ARGs were annotated. Based on the functional level of Drug Class, the most significant antibiotic types for ARGs resistance were aminoglycoside antibiotic (45 kinds), glycopeptide antibiotic (36 kinds of glycopeptide antibiotics), tetracycline antibiotic (tetracycline antibiotics, 31), and phenicol antibiotic (phenicol antibiotics, 18). The tetracycline antibiotic, which had the highest relative abundance, exhibited no significant difference between the two groups during the four time points (approximately 24–29%). The content of glycopeptide antibiotic was the second highest, and at 21d and 28d, it was significantly higher in the FM group than in the BM group (21d: BM: 15.5%, FM: 21.05%, *p* = 0.0084; 28d: BM: 20.76%, FM: 28.76%, *p* = 0.0227) (Supplementary Figure S7A and Supplementary Table S8).

Considering that the gene for anti-Glycopeptide antibiotic was significantly enriched in the FM group at 21d and 28d, LEfSe analysis of related ARGs in the two groups was conducted. At 21d and 28d, vanG, vanTG, vanYB, and vanHO were identified to have the greatest contribution to glycopeptide antibiotic resistance (Supplementary Figures S7B,C). ARGs of the van series were mainly associated with vancomycin resistance. Vancomycin is a broad-spectrum antibiotic belonging to the glycopeptide class, which is mainly employed to treat serious infections caused by Gram-positive bacteria (Rybak, 2006), such as Methicillin-resistant *Staphylococcus aureus* (MRSA). Although no direct correlation between formula milk components and vancomycin resistance has been reported, our findings from the preceding section of this study showed that GH2 and GH20 were significantly higher in the FM group compared to the BM group. GH2 and GH20 may influence antibiotic glycosylation, and the metabolic products (such as glucuronic acid and N-acetylglucosamine) released from the breakdown of host or drug glycosides could act as signaling molecules (Wardman et al., 2022). These molecules might activate bacterial quorum sensing systems or oxidative stress responses, promoting the horizontal transfer of resistance genes, which could be one of the factors influencing the prevalence of resistance genes. The elevated prevalence of these ARGs within the FM group could render the treatment of MRSA and other drug-resistant bacterial infections in pig farming more challenging, augmenting the medical costs and mortality rate in pig breeding (Murray et al., 2022). Hence, it is essential to pay heed to the prevention and treatment of MRSA and other drug-resistant bacterial infections in pig farming by avoiding the application of this class of antibiotics in artificial formula milk feeding.

## 4 Conclusion

In this study, the disparities of the fecal microbiota in SPF Bama pigs were analyzed under breastfeeding (BM group) and formula milk artificial feeding (FM group), and the impacts of different feeding methods on the gut microbiota community structure and function of SPF Bama pigs were disclosed. Through comprehensive analyses of microbiota diversity, dominant microbiota, functional metabolism, CAZymes, and ARGs, we concluded that there were significant differences in the effects of breastfeeding and formula feeding on the gut microbiota community of SPF Bama pigs. While breastfeeding contributes to the maintenance of gut microbiota diversity and balance, the formula-fed group demonstrated higher activity in galactose metabolism and glycan metabolism, especially at 21d, with the most pronounced differences between the two groups. At the same time, several potential microbiota that might have an influence on glycan metabolism were also identified. Regarding ARGs, ARGs of glycopeptide resistance were significantly enriched in the formula-fed group at 21d and 28d. The findings of this study offer novel insights into how different feeding patterns shape the gut microbiota of pigs and provide a scientific basis for optimizing pig feeding strategies and enhancing breeding efficiency. Additionally, these findings may also have significant practical applications for the pig breeding and husbandry industry. Future research could further explore how specific components in breast milk and formula affect the development and function of the gut microbiota and how these changes impact the growth performance and health of pigs.

## Data Availability

The datasets presented in this study can be found in online repositories. The names of the repository/repositories and accession number(s) can be found at: https://www.ncbi.nlm.nih.gov/, SRP548277.

## Footnotes

^1^ https://www.kegg.jp/ghostkoala/

^2^ https://www.bioincloud.tech/
