# Supplementary figures and images for: Dynamic changes in the gut microbiota of SPF Bama piglets during breast and formula feeding
Source: Front Microbiol. 2025 Feb 26;16:1537286. doi: 10.3389/fmicb.2025.1537286 (PMC11897505; doi:10.3389/fmicb.2025.1537286)

**birth lenth**

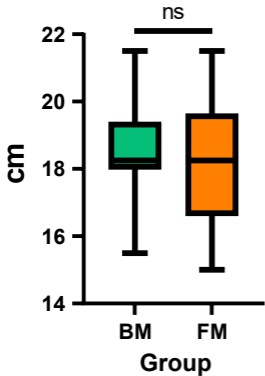

**birth weight**

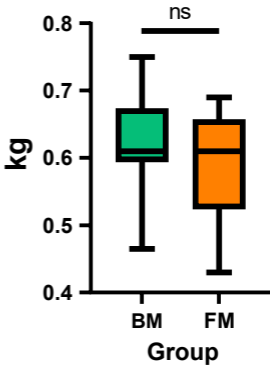

Supplement: Supplementary file 1 [file Data_Sheet_1.pdf]

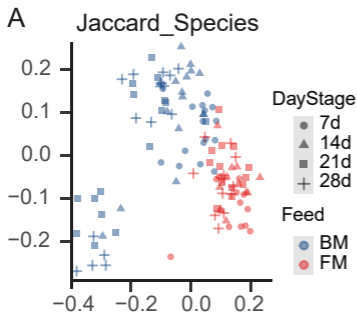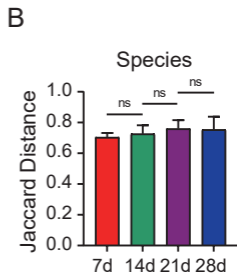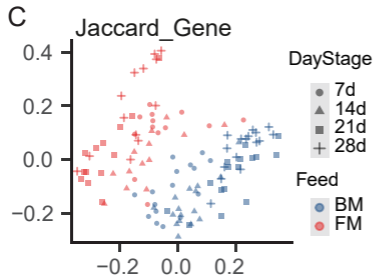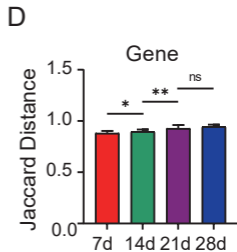

Supplement: Supplementary file 2 [file Data_Sheet_2.pdf]

A

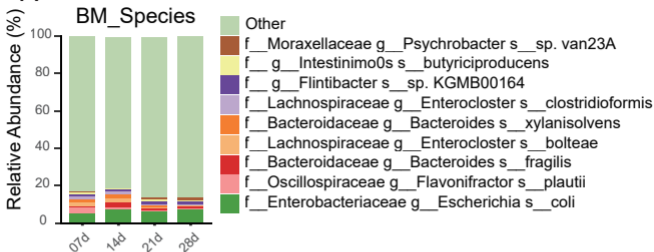

B

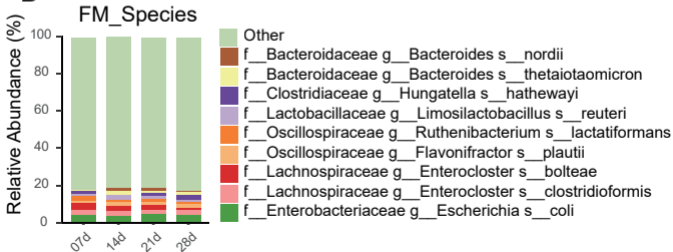

Supplement: Supplementary file 3 [file Data_Sheet_3.pdf]

7d

Species

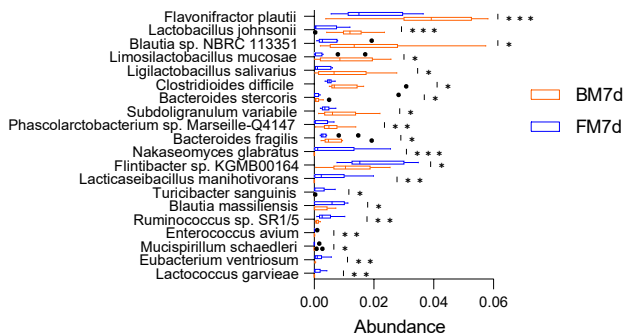

21d

Species

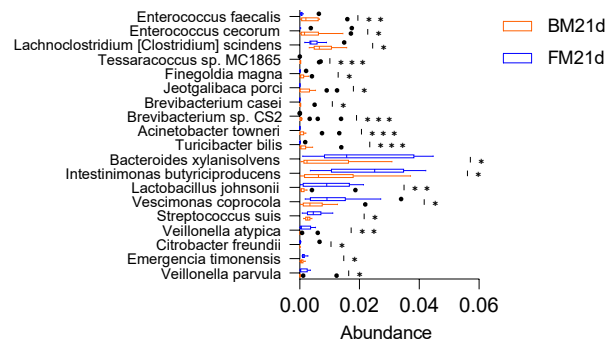

28d

Species

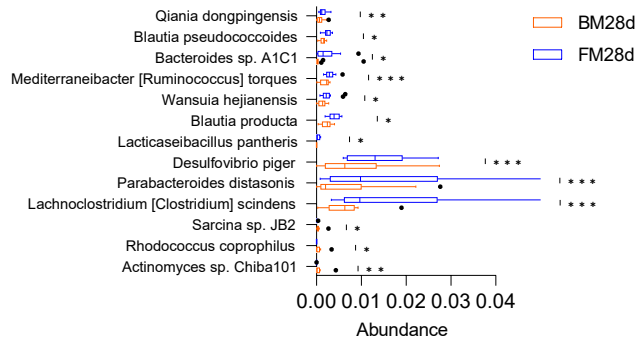

21-28d\_shared\_21d

Species

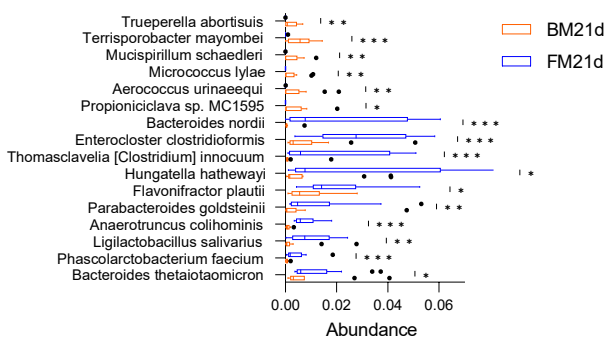

21-28d\_shared\_28d

Species

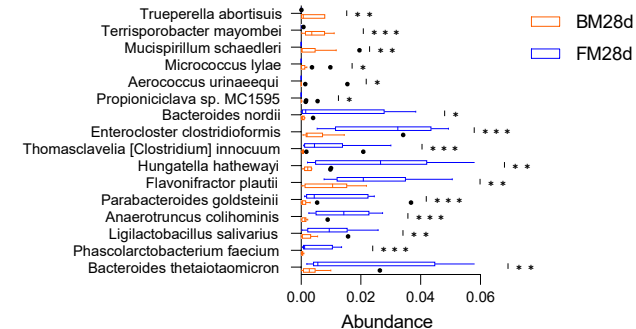

Supplement: Supplementary file 4 [file Data_Sheet_4.pdf]

7d

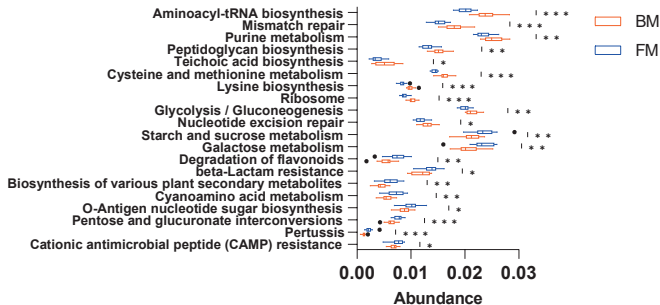

14d

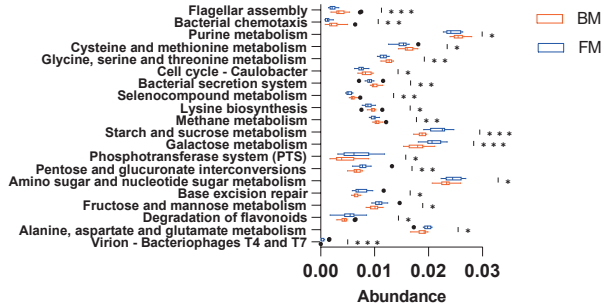

21d

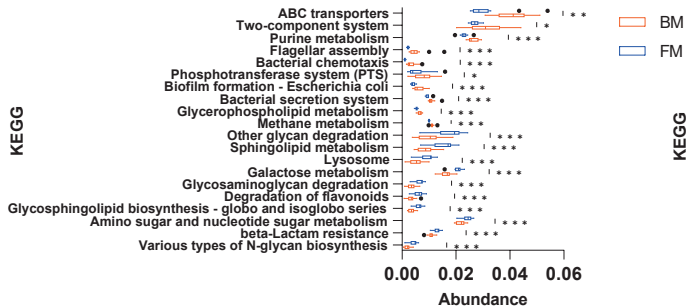

28d

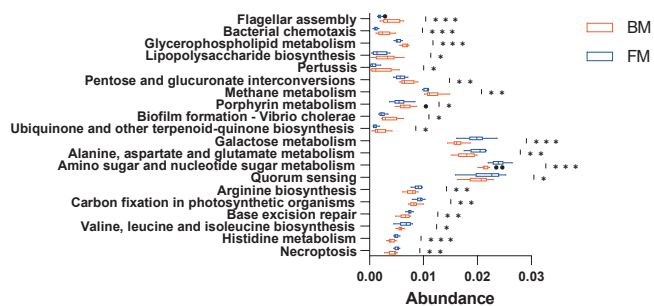

Supplement: Supplementary file 5 [file Data_Sheet_5.pdf]

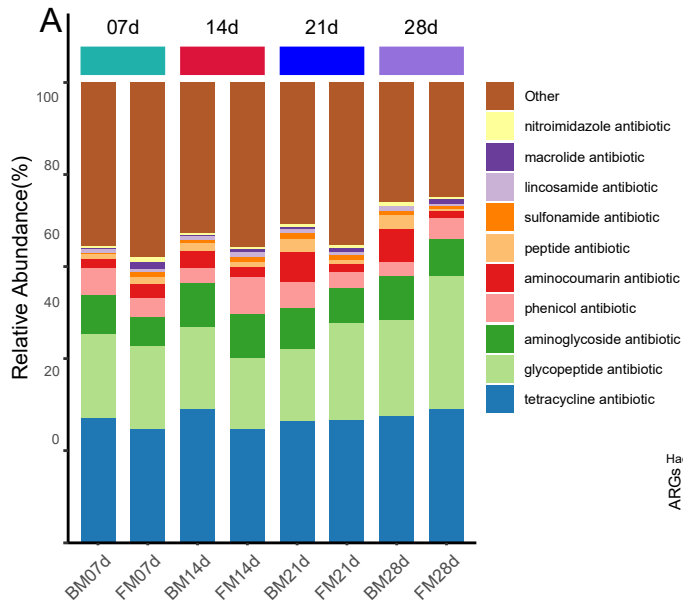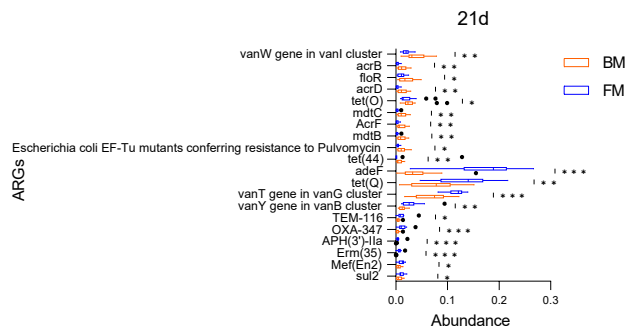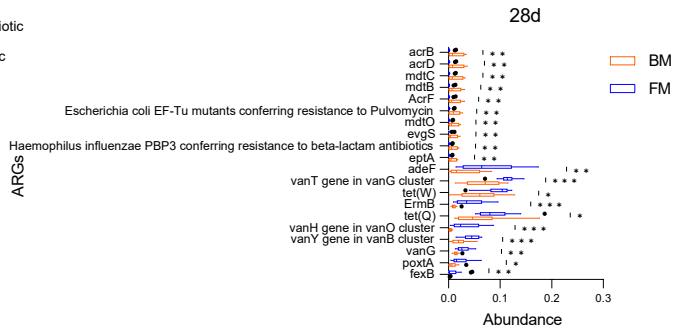

Supplement: Supplementary file 7 [file Data_Sheet_7.pdf]
