# Supplementary material for: Dynamic changes in the gut microbiota of SPF Bama piglets during breast and formula feeding
Source: Front Microbiol. 2025 Feb 26;16:1537286. doi: 10.3389/fmicb.2025.1537286 (PMC11897505; doi:10.3389/fmicb.2025.1537286)
Supplement: Supplementary file 8 [file Data_Sheet_8.docx]

Supplementary Material

Dynamic Changes in the Gut Microbiota of SPF Bama Pigs Under Different Feeding Methods

Chengcheng Zhang1†, Zhengjiang Liu3† , Huan Yu1, YuanYuan Shen1, Lu Lu1, Fanli Kong3, Wei Sun4, Xiaoyuan Wei5, Long Jin12, Liangpeng Ge,6* Bo Zeng12*

*** Correspondence:**Bo Zeng, apollobovey@163.com

geliangpeng1982@163.com

**Data availability statement**

All sequencing data are available in the NCBI Sequence Read Archive (SRA) under the bioproject number PRJNA1191908, submission: SUB14868929.

# Supplementary tables

Table S1. Sample information

Table S2. Formula milk ingredients

Table S3. The influence of sample background factors on beta diversity

Table S4. Beta diversity significance test of microbiota

Table S5. Significance test of dominant bacterial phylum communities

Table S6. Significance test of dominant bacterial communities at different time points

Table S7. Gut microbial Species correlated to CAZyme

Table S8. Significance test of dominant DrugClass of ARGs between BM and FM group

# Supplementary figures

Figure S1. a.Boxplot displays the initial birth weight of the BM group and the FM group. b.Boxplot displays the initial birth length of the BM group and the FM group.

Figure S2. a. Jaccard distance matrix of species is presented on the principal coordinate analysis (PCoA) plot. b. The difference in Jaccard distance in terms of species between the BM group and the FM group at the same time points is shown. c. The Jaccard distance matrix of genes is presented on the principal coordinate analysis (PCoA) plot. d. The difference in Jaccard distance in terms of genes between the BM group and the FM group at the same time points is shown.

Figure S3. Bar plot is presented to represent the relative abundance of dominant species at four time points within the a.BM and b.FM groups at the Species level.

Figure S4. a-c. Boxplot is presented the the relative abundance for the LEfSe analysis of unique differential species to each group at three time points(a.7d, b.21d,c. 28d). d-e. Boxplot is presented to represent the relative abundance of differential species shared by both groups from 21d to 28d(d.21d, e.28d). In each boxplot, the upper part represents species with significantly higher abundance in the BM group compared to the FM group, while the lower part represents species with significantly higher abundance in the FM group compared to the BM group. Each group is sorted in ascending order according to LDA.

Figure S5. Boxplot is presented the the relative abundance for the LEfSe analysis of diffrential KEGG pathway in two groups at 4 time points(a.7d, b.14d,c.21d, d.28d). The horizontal axis represents the relative abundance, and the vertical axis represents the names of KEGG pathway. In each boxplot, the upper part represents KEGG pathway with significantly higher abundance in the BM group compared to the FM group, while the lower part represents KEGG pathway with significantly higher abundance in the FM group compared to the BM group. Each group is sorted in ascending order according to LDA.

Figure S6. Network diagram of the correlation between species and CAZymes. The relationship coefficient r > 0.5, edges=green. Node: Species=red, PLs=green, GHs=purple.

Figure S7. a. Stacked bar chart is presented to represent the relative abundance of the dominant Drug Class of ARGs in the two groups at the 4 time points. The vertical axis represents the proportion of Drug Class in the sample. Different colored columns signify different Drug Class, and the length of the columns represents the proportion of Drug Class. b-c. Boxplot is presented the the relative abundance for the LEfSe analysis of diffrential ARGs in two groups at 2 time points(a.21d, b.28d).
