# Supplementary material for: Dynamic changes in the gut microbiota of SPF Bama piglets during breast and formula feeding
Source: Front Microbiol. 2025 Feb 26;16:1537286. doi: 10.3389/fmicb.2025.1537286 (PMC11897505; doi:10.3389/fmicb.2025.1537286)
Supplement: Supplementary file 6 [file Data_Sheet_6.pdf]

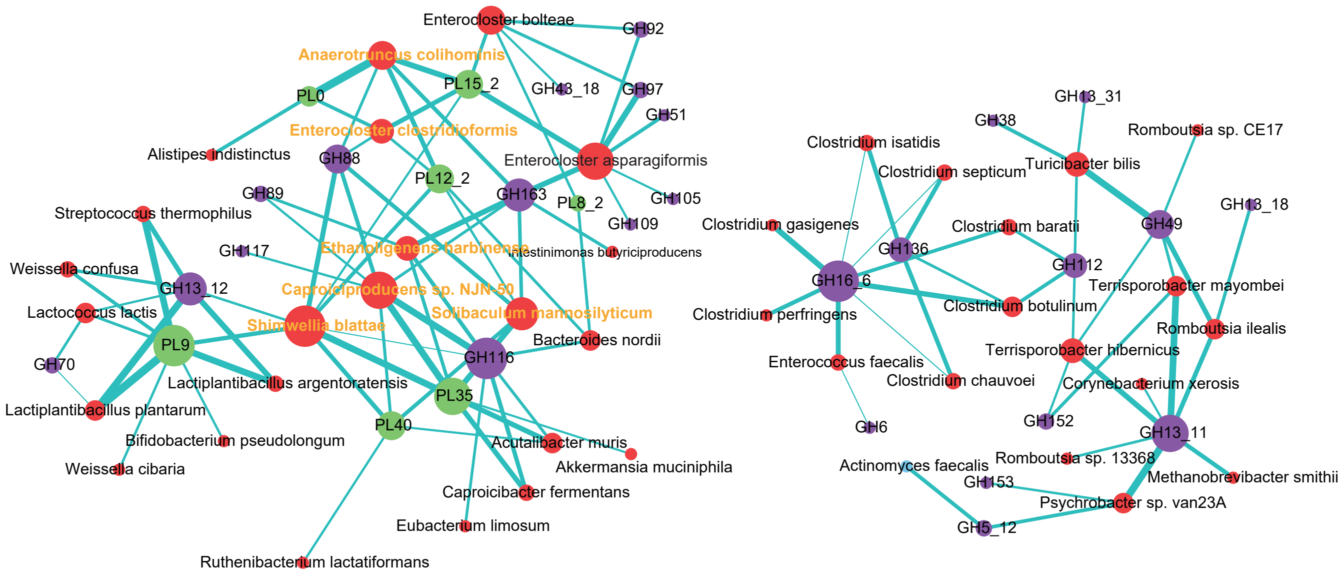
Enterocloster bolteae
GH92
Anaerotruncus colihominis
PL0
PL15_2
GH43_18
GH97
GH51
Enterocloster clostridioformis
Alistipes indistinctus
GH88
PL12_2
Enterocloster asparagiformis
GH89
GH163
PL8_2
GH105
GH109
Streptococcus thermophilus
GH117
Ethanoligenens harbinense
Intestinimonas butyriciproducens
Weissella confusa
GH13_12
Caproiciproducens sp. NJN-50
Lactococcus lactis
Solibaculum mannosilyticum
Shimwellia blattae
PL9
Bacteroides nordii
GH70
GH116
Lactiplantibacillus argentoratensis
PL35
Lactiplantibacillus plantarum
PL40
Bifidobacterium pseudolongum
Acutalibacter muris
Weissella cibaria
Akkermansia muciniphila
Caproicibacter fermentans
Eubacterium limosum
Ruthenibacterium lactatiformans
GH13_31
GH38
Romboutsia sp. CE17
Clostridium isatidis
Turicibacter bilis
Clostridium septicum
GH13_18
Clostridium gasigenes
Clostridium baratii
GH49
GH136
GH112
GH16_6
Terrisporobacter mayombei
Clostridium perfringens
Clostridium botulinum
Romboutsia ilealis
Terrisporobacter hibernicus
Enterococcus faecalis
Clostridium chauvoei
Corynebacterium xerosis
GH152
GH6
GH13_11
Actinomyces faecalis
Romboutsia sp. 13368
GH153
Methanobrevibacter smithii
Psychrobacter sp. van23A
GH5_12
